# Moving Together: Social Motor Synchrony in Autistic Peer Partners Depends on Partner and Activity Type

**DOI:** 10.1007/s10803-023-05917-8

**Published:** 2023-06-13

**Authors:** Devyn Glass, Nicola Yuill

**Affiliations:** University of Sussex, Brighton, BN1 9QJ England, UK

**Keywords:** Social motor synchrony, Autism spectrum conditions, Social interaction, Peer interaction, Digital technology, Motion energy analysis

## Abstract

Some suggest autistic people display impaired Interpersonal Synchrony. However, partners of different neurotypes can struggle to connect and empathise with one another. We used Motion Energy Analysis to examine Social Motor Synchrony (SMS) in familiar partners of the same neurotype: pairs of autistic and of neurotypical children. Partners played two shared tablet activities, one to support collaboration by facilitating engagement and other-awareness (Connect), and one with no additional design features to facilitate collaboration (Colours). The neurotypical group showed similar SMS to the autistic group in Colours but lower SMS in Connect. The autistic group displayed similar levels of SMS in each activity. Autistic children can synchronise to a similar, or greater, degree than neurotypical children when the social context and type of task are considered.

Autism Spectrum Condition (ASC) is clinically defined as a neurodevelopmental condition characterised by difficulties in social interaction and communication, sensory and cognitive processing, and restricted or repetitive behaviours or interests (American Psychiatric Association, 2013). There is an ongoing shift in the conceptualisation of autism towards a model of neurodivergence, which considers the atypicality in social interaction and cognition often associated with autism, as differences rather than deficits (Jurgens, 2020; Kapp, 2020). Autism is increasingly recognised as a different ‘disposition’, and autistic people are said to perceive and experience the world differently than non-autistic, or ‘neurotypical’ (NT) people do (Milton, 2014). We refer to autism as a ‘condition’, rather than a ‘disorder’, and use both identity- and person-first language. This reflects a move towards a neurodiversity paradigm and aligns with recent research indicating the varied preferences of the autistic community (Buijsman et al., 2022; Bury et al., 2022).

One defining feature of autism commonly used in diagnosis is a difficulty with social interaction and communication. Some suggest a difficulty in the ability to synchronise behaviours and movements with others may underlie some social interaction differences (e.g., Fitzpatrick et al., 2016). When achieved in social interaction, Interpersonal Synchrony (IPS) involves coordinated interpersonal movement, with an element of “rhythmically matched timing” (Tarr et al., 2018, p. 1). IPS involves a range of social communicative exchanges, such as joint attention, turn-taking, shared affect, or engagement (Charman, 2011), and is frequently associated with measures of rapport and social connectedness (Lakens & Stel, 2011; Miles et al., 2009; Tschacher et al., 2014). A recent body of literature has focused on a specific element of IPS: Social Motor Synchrony (SMS), which refers to simultaneous or rhythmically matched body movements (Fitzpatrick et al., 2016). Specifically, this body of literature is concerned with the synchrony of non-verbal and non-facial social communicative exchanges (Fitzpatrick et al., 2016). Several studies have shown that pairs involving an autistic partner (i.e., two autistic partners, or one autistic and one NT partner) show less SMS in their social interactions compared with two NT partners (e.g., Fitzpatrick et al., 2017; Georgescu et al., 2020; Zampella et al., 2020) Many researchers therefore subscribe to an SMS model of autism, which suggests autistic people show impaired SMS, and where lower SMS is said to underlie difficulties with social interaction and communication.

Most SMS studies involving autistic people compare interactions between matched-NT pairs (two neurotypical partners) and mixed pairs (one autistic and one NT partner), finding lower SMS in mixed pairs than in matched-NT pairs (Glass & Yuill, 2023; Mcnaughton & Redcay, 2020). These group differences have resulted in the attribution of misattunement and disruptions in synchrony to the autistic person in a mixed pair. However, social relationships and interactions involve reciprocity and mutuality. Social partners have a shared understanding of the norms and expectations of their interaction (Milton, 2012; Petrina et al., 2014). Milton (2012) emphasises that these social expectations are co-constructed within the interaction, and when social partners have different dispositions and experiences of the world, difficulties with social interaction emerge. This has been termed the Double Empathy Problem, where both autistic and non-autistic people can struggle to understand or empathise with one another (Milton, 2012). Bolis et al. (2018) refer to this experience as dialectical misattunement. They argue that social partners with different interaction styles can be interpersonally mismatched, resulting in less smooth and socially synchronous interactions than when two partners are interpersonally similar (Bolis et al., 2018). When studying SMS in autism, we should therefore consider the embodied and bidirectional nature of social interaction and variability in social dispositions. Including matched-autistic dyads is one way to examine SMS in dyads with similar social dispositions.

So far, only one SMS study involved all three potential dyad types: matched-autistic, matched-NT, and mixed adult partnerships (Georgescu et al., 2020). Their findings showed significantly lower SMS in matched-autistic pairs and mixed pairs compared with matched-NT pairs during a series of conversation tasks (Georgescu et al., 2020). Additionally, Stoit et al. (2011) found matched-autistic pairs displayed less synchrony than matched-NT pairs during a cooperative balancing task. While the limited research so far lends support to an SMS model of autism, more research is needed involving matched-autistic dyads to better understand SMS in autism. This is especially important considering the growing literature detailing the accounts of autistic people feeling comfortable in interactions with other autistic people, and more connected than they do when interacting with NT partners (Crompton et al., 2020b, 2020c). For instance, matched-autistic pairs reported more rapport than partners did when interacting with someone from a different neuro-type (Crompton et al., 2020a). Bolis et al. (2021) demonstrated that the more similar autistic people were in ‘autistic traits’, the closer they perceived their friendship. Additionally, Williams et al. (2021) found matched-autistic and matched-NT pairs communicated more fluidly, with stronger rapport and intersubjectivity than mixed pairs. Given the role of social connectedness in IPS (Lakens & Stel, 2011; Miles et al., 2009; Tschacher et al., 2014), if communication is smoother and interactions are more comfortable between two autistic people, we may expect further research to show SMS in matched-autistic pairs to be higher than mixed pairs in some contexts.

There are elements in the SMS tasks used so far that may account for the lower SMS we have seen in matched-autistic dyads. SMS studies typically use either intentional or spontaneous tasks. The former includes tasks such as intentionally synchronising movements to an experimenter or with an object, such as a pendulum (e.g., Fitzpatrick et al., 2016, 2017), or moving a virtual bar (Stoit et al., 2011). These constrained tasks require planned movement, which is dependent on additional cognitive processes. We know some autistic people can have executive function difficulties (Craig et al., 2016; Demetriou et al., 2017), which might disrupt synchronous interactions with a partner during cognitively-demanding tasks or contexts. Other SMS tasks, such as building a puzzle or synchronising body movements to an experimenter (Delaherche et al., 2013; Fitzpatrick et al., 2017) involve cognitive processes known to be difficult for some autistic people, such as attention, working memory, and movement planning (Vaidya et al., 2020).

Spontaneous and intentional synchrony are separate constructs (Fitzpatrick et al., 2018) and autistic people show synchrony with their partners in some tasks that allow for spontaneous SMS. Ward et al. (2018) illustrated how measuring SMS during open and dynamic activities can detect subtle moments of synchrony between different dyad types, including between autistic siblings and autistic children and drama facilitators. Interactions between practitioners and autistic children also yield close synchrony during Dance and Movement Therapy, with SMS increasing over time (e.g., Dvir et al., 2020; Koehne et al., 2016). Spontaneous synchrony may therefore be higher for autistic people and their partners than intentional synchrony owing to the absence of additional cognitive demands.

Autistic people are also known to possess more focused attention than NT people, often relating to specific topics of interest, an experience termed monotropism (Murray, 2018; Murray et al., 2005). Studies with tasks designed to be meaningful for autistic participants or with content aligned to their interests have revealed rich and reciprocal interactions (e.g., Williams et al., 2021). Video games are popular and engaging for many autistic people, who describe them as beneficial for social connection (Mazurek et al., 2015), and often select them as a preferred activity (Heasman & Gillespie, 2019). Sitting side-by-side might also be more comfortable for autistic people than sitting opposite one another, as is common in other SMS tasks (Heasman & Gillespie, 2019). Technology is frequently used in a learning environment to retain the attention and engagement of pupils with autism (Correia & Halabi, 2021) and to support skills that are associated with SMS, such as social interaction (e.g., Alcorn et al., 2013) and collaboration (e.g., Holt & Yuill, 2017). A benefit of digital tools is the potential to design the content with the needs and preferences of an autistic participant or learner in mind. Personalisation of content can include specialised interests, which can increase social motivation, and they can be designed to constrain for certain behaviours, such as awareness of a partner (Davey, 2020; Wilson et al., 2017; Yuill, 2021). Some activities also allow for simultaneous gameplay and foster responsiveness to a partner’s actions, which provide additional avenues for SMS (Holt & Yuill, 2017; Marsh et al., 2009).

In sum, previous literature has found lower SMS in social interactions in matched-autistic pairs compared with mixed pairs or pairs of NT people (e.g., Delaherche et al., 2013; Georgescu et al., 2020; Marsh et al., 2009). This has resulted in the proposal of an SMS model of autism, in which autistic people are said to display impaired SMS. However, synchrony involves mutuality and is a property of an interaction, not an individual trait. It is therefore important to consider the dynamics of the social context, such as the impact of pairing participants of different neuro-types or dissimilar social dispositions. We therefore aim to examine Social Motor Synchrony (SMS) in pairs of autistic children compared with pairs of neuro-typical (NT) children when partners are of the same neuro-type, are familiar with one another, and have been matched according to their relationships and perceived disposition. We will use the affordances of tablet technology to tailor activities for autistic participants to be socially motivating and to support reciprocal social interactions (Davey, 2020; Heasman & Gillespie, 2019). We will examine whether a specially designed tablet activity (Connect), which is personalised and tailored to support collaboration and engagement in children with autism, facilitates SMS compared to shared tablet activity with no additional design features to support collaboration (Colours).

## Method

This study was approved by the University’s Sciences and Technology Cross-Schools Research Ethics Committee and was conducted in a mainstream school and a special education school. Written informed consent for the children and young people to take part and be video-recorded was obtained from parents/carers prior to the study days. The children gave their assent to take part and to be video recorded on the day.

### Participants

A total of 25 children and young people participated (see Table 1). The autistic group included 13 participants (1 female, 12 male) aged 6–13 years (*M* = 9.24, *SD =* 2.28) from a UK special education school. All had autism diagnoses recorded on Education and Health Care Plans.^1^ Eight had additional diagnoses of speech and language difficulties and one had diagnoses of global developmental and mild cognitive delay. Six of the autistic children with additional diagnoses were described by parents and teachers as having limited verbal abilities. The remaining seven used verbal communication as their primary method of communication. The neuro-typical (NT) group included 12 participants (6 female, 6 male) aged 6–9 years (*M* = 8.28, *SD =* 0.99) from a UK mainstream school. No NT participants had diagnoses or special educational needs reported by parents or school.

**Table 1:** Demographic data for the autistic and neuro-typical groups

Group	Gender (F:M)	Age M (SD)	AQ M (SD)	Social Responsiveness Scale Scores M(SD)					
				Total SRS	Social awareness	Social cognition	Social Comm.	Social motivation	RRB
Autistic	1:12	9.24 (2.28)	7.31 (1.32)	80.77 (8.62)	79.62 (8.1)	77.92 (9.4)	75.62 (9.73)	71.77 (10.73)	80.77 (8.62)
NT	6:6	8.28 (0.99)	NA	46.75 (6.06)	45 (10.34)	44.17 (6.49)	44.33 (6.51)	44.75 (5.71)	46.75 (6.06)

*NT* neuro-typical, *AQ* autism quotient, *SRS* Social Responsiveness Scale, *Social Comm.* social communication, *RRB* restrictive and repetitive behaviour

Teachers of the autistic children completed the child version of the AQ-10 (Allison et al., 2012) as an additional indicator they met the threshold at which they would usually be considered for an autism assessment. Usually, a score of 6 or above indicates the child may be autistic. We removed one item which referred to behaviour at pre-school as current teachers would not have known the child then. We took a score of 5 on the reduced scale as our autism screening threshold, and all autistic participants scored above this. Parents of both autistic and NT children completed the Social Responsiveness Scale (SRS-2, Constantino & Gruber, 2014), except for two autistic students, for whom the SRS was completed by teachers. This was to distinguish the autistic and NT groups and consider whether synchrony is associated with social difficulties and autistic traits. The SRS-2 is sensitive to autistic traits in the general population, with higher scores indicating more autistic traits (Constantino & Gruber, 2014). There was a significant difference in SRS scores between the groups (*t*(23) = 11.16, *p* < .001, *d* = 4.47), with the autistic group (*M* = 80.08, *SD* = 8.83) scoring significantly higher than the NT group (*M* = 44.33, *SD* = 7).

### Materials and Procedure

**Fig. 1:**
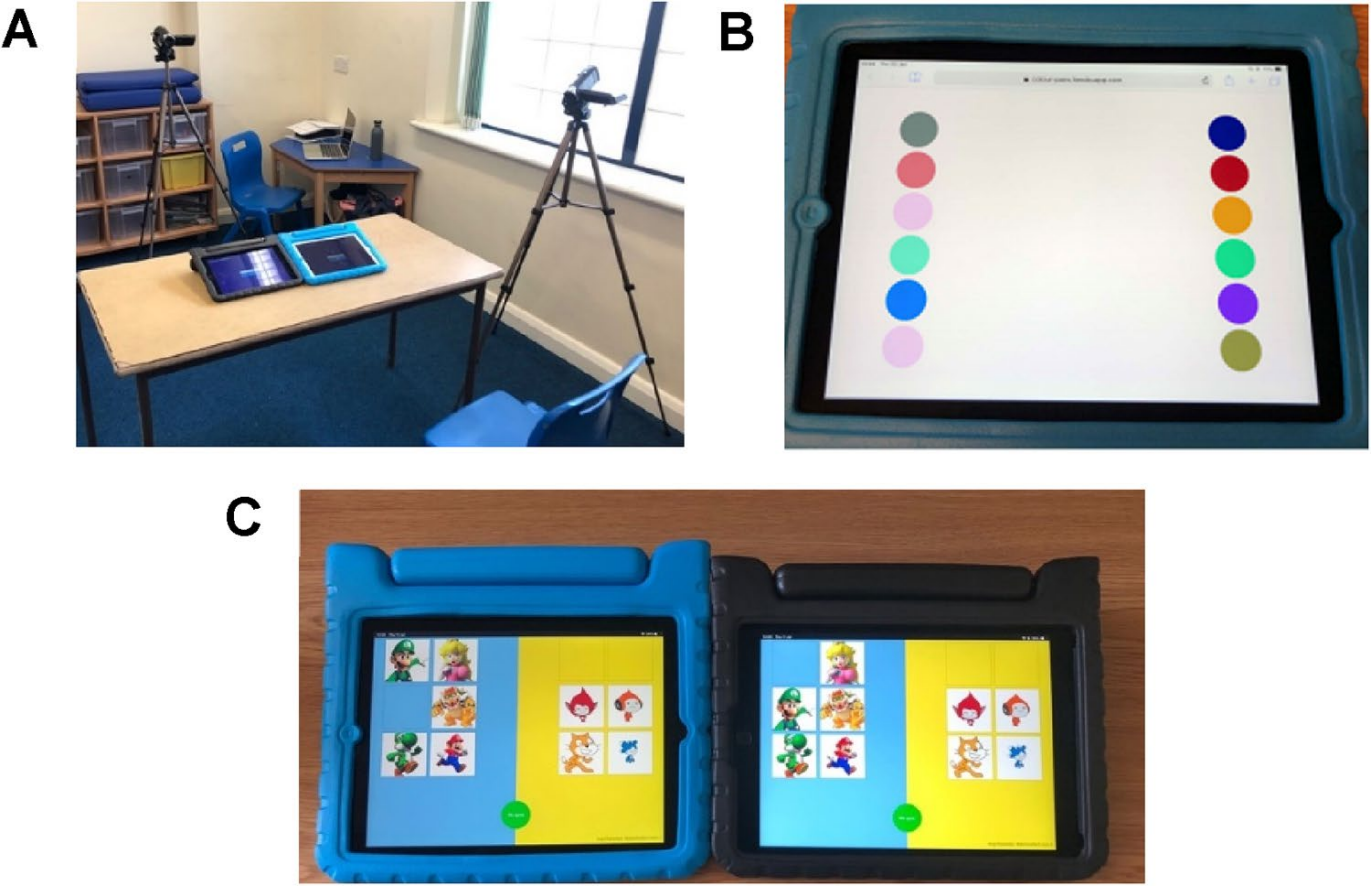
**a** The classroom recording set-up where pairs of children played the two collaborative tablet activities. **b** The Colours app, played on a single, shared tablet device. **c** The Connect App, played on dual, connecting tablets

Participants took part in pairs during the school day. Partners were selected by the teachers according to their compatibility. The autistic group consisted of one mixed gender pair and six pairs with partners of the same gender (boys). The NT group consisted of six mixed gender pairs as they were selected from the same class and were paired by the teacher, who judged the partners would get along well. All pairs were of the same neurotype and were familiar with one another, having been in the same school class for at least one academic year. One autistic participant did not complete Connect and chose to leave before Colours, so their partner was allocated a new partner, as parental consent was obtained for an odd number of autistic pupils. The new pair were unable to play Connect due to technical difficulties. One other pair completed only Connect. Therefore, four pairs of autistic children played both activities, two played Connect only, and one played Colours only. All NT children played both activities. Pairs sat side-by-side to be recorded by two static cameras while playing the activities on iPads (see Fig. 1a). Pairs were randomly assigned to complete either Colours or Connect first.

#### The Colours App

Colours is a web app, which hosts a simple colour-matching activity played on a single tablet (see Fig. 1b). Players are required to find the one matching pair of coloured dots, which appear in columns on either side of the tablet. The difficulty increases with each round as the number of dots increases. The two columns align with where each child sits but the children’s attention was not drawn to the two seemingly distinct sides. Pairs were given a single shared tablet and were encouraged to work together to find the matching pair.

#### Connect

Connect is another web app, which hosts a picture-sorting activity played across two adjacent tablets. It is designed to support awareness of a partner and contingent behaviour (Holt & Yuill, 2017). The aim of Connect is to stimulate interaction with a partner and shared understanding of the task, rather than rapid completion of the picture-sorting. Tablets are connected via Wi-Fi, creating a connected activity where users work together to place pictures into cells (see Fig. 1c). Pictures are ‘matched and sorted’; each player must have their picture in the same location as their partner’s picture, and they must be grouped correctly according to two pre-defined categories. For instance, in Fig. 1c. Super Mario Bros® characters would be grouped on one side, and Scratch® characters would be grouped on the other side. Connect was personalised to the interests of each autistic pair and included images such as Minions characters, Super Mario Bros® characters, food, and sensory objects. For the NT children, the content was tailored to align to their curriculum and was designed to be slightly more challenging. They were asked to sort pictures of animals into ‘sea’ and ‘land’ animals, with some ambiguous items included to spark discussion.

### Analyses

**Fig. 2:**
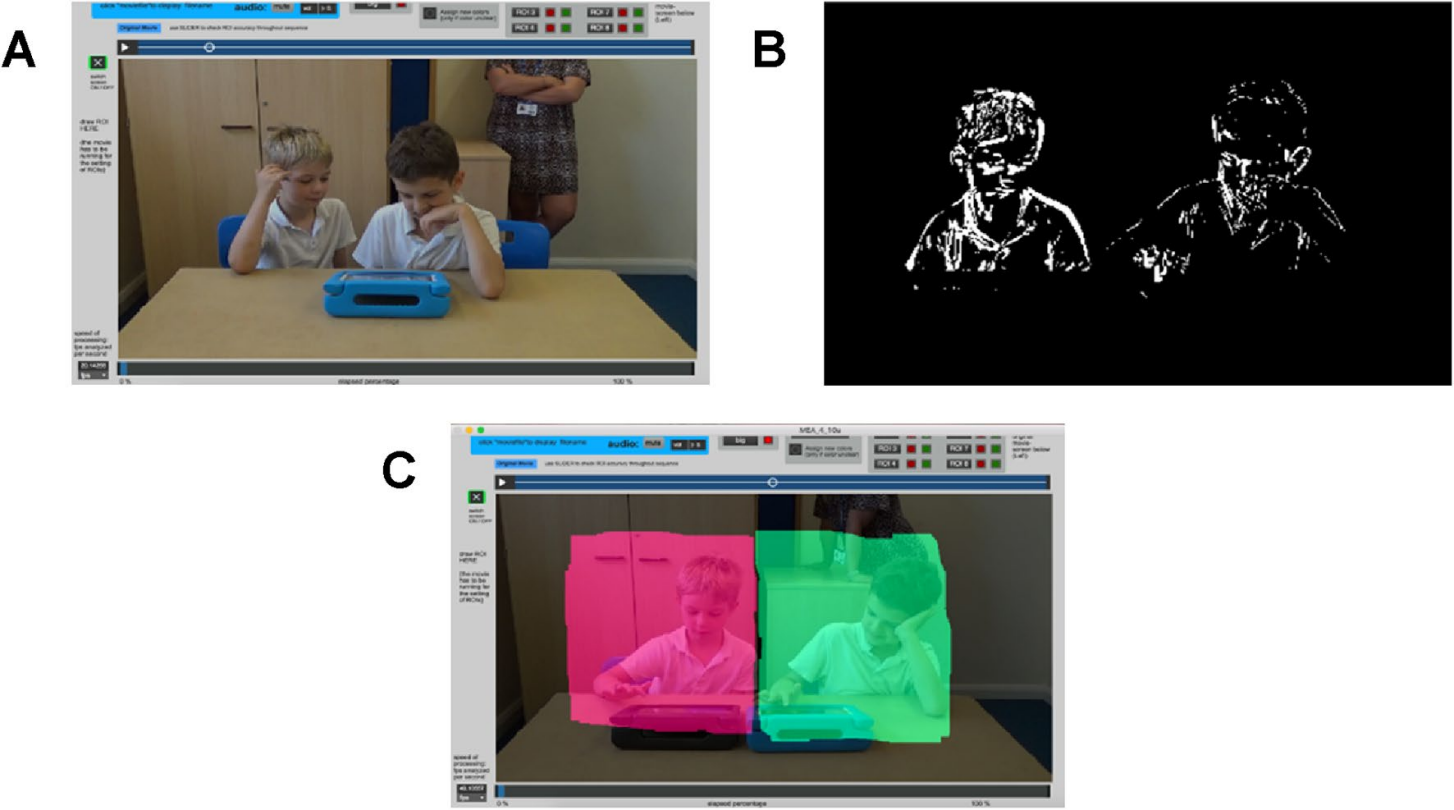
**a** Still image of one video recording imported into the MEA software. **b** Image demonstrating the pixel change captured by the MEA frame differencing method. **c** Still image of one video recording with pre-defined regions of interest for partner 1 (left of the image) and partner 2 (right of the image). *Parental consent was granted for the use of images*

We used Motion Energy Analysis (MEA) to compare Social Motor Synchrony (SMS) in autistic compared to NT pairs in the single and dual-tablet activities (Kleinbub & Ramseyer, 2020; Ramseyer, 2020). MEA is an automated procedure to measure movement from a video recording (see Fig. 2a). It uses a Frame Differencing Method to monitor changes in pixels frame-by-frame (see Fig. 2b). Each partner’s motion is captured separately by pre-defining regions of interest (ROIs). As participants in this study were seated side-by-side at a table, we used one ROI per participant, which captured the motion of their arms, torso, and heads (see Fig. 2c). The MEA programme is not able to identify when one partner crosses into the other partner’s ROI (Ramseyer, 2020). It is therefore important that recordings do not include clips of partners moving in front of one another. In instances where participants crossed into their partner’s ROI, such as pointing toward a picture on their partner’s iPad, small segments of the video were removed prior to extracting the MEA data. The cut segments were no longer than 3 seconds and less than 10 were taken from each video. The resulting data were two continuous time-series showing the amount of movement by each partner.

To quantify synchrony in participant dyads, we used the rMEA package for R Studio (Kleinbub & Ramseyer, 2020). Using the time-series extracted from MEA, we calculated windowed and lagged cross-correlations (WLCC) for the two partners in a dyad. A cross-correlation is a measure of similarity of two time-series. WLCC splits the video into segments, or windows, meaning the strength of the correlation can vary across the time-series, thus allowing us to account for the changeable nature of synchrony during interaction (Boker et al., 2022; Roume et al., 2018). Time-lagged synchrony enables synchronous movement to be identified when there is a delayed onset, such as when one partner initiates a movement, acting as a leader, and the second partner follows or joins in, creating synchronous motion. The value can be positive or negative as a means of identifying which partner is leading and when there is a lag (Boker et al., 2002). We calculated time-lagged synchrony between partners with a maximum lag of ± 5 seconds and selected windows of 10 seconds with increments of 2 seconds. This means the cross-correlations were calculated in steps of 2 seconds and were performed discretely in every 10-second window. We used these parameters to capture brief and micro-level synchrony, which was characteristic of this dataset (Kleinbub & Ramseyer, 2020). The SMS score was computed by standardising the cross-correlations using Fisher’s *Z* transformation, which accounts for different-sized ROIs (due to different sized bodies or different sized spaces in which the partners moved; Ramseyer & Tschacher, 2011), and their absolute values were combined over the whole video to give one overall SMS value. The absolute value contains positive and negative cross-correlations, which means both in-phase and anti-phase correlations positively contributed to the overall measure of synchrony. To compare SMS scores in the autistic compared with the NT group across the two activities, and to examine within-group differences in SMS according to the activity type, we computed a two-way ANOVA.

Windowed cross-correlations alone provide no control for coincidental synchrony, which makes it difficult to determine whether the synchrony has occurred due to attunement between the two partners or due to chance (Ramseyer & Tschacher, 2011). Consequently, we computed pseudo-synchrony scores by pairing a single time-series collected in one dyad with a single time-series collected from a different dyad to create SMS scores between two partners who did not interact. To do this, we followed Kleinbub & Ramseyer’s (2020) shuffling procedure, which reorganises the time-series into different pairs to create a random set of dyads. We calculated synchrony in the pseudo-dyads using the method described for computing synchrony in the real dyads, then compared the groups using t-tests to examine whether the SMS displayed by the real dyads significantly differed from the SMS we would see by chance, represented by the pseudo-dyads’ SMS scores.

We also extracted average Motion Energy scores to rule out the possibility that any synchrony differences between groups was due to differences in the amount each group moved. The degree of motion energy in the autistic group was not normally distributed. We therefore used a Wilcoxon Rank Sum test to compare average Motion Energy in the autistic and NT groups and a Spearman correlation to examine whether average Motion Energy was associated with SMS. Systematic differences in movement quantity between groups could influence SMS scores and an association between average Motion Energy and SMS would caution the interpretation of results (Georgescu et al., 2020).

Finally, we computed a series of Pearson correlations to examine whether SMS scores were related to autistic traits. We examined the association between (a) SMS scores and mean SRS scores in each group, and (b) SMS scores and an SRS difference score. Computing an SRS difference score enabled us to examine whether higher SMS scores were related to interpersonal similarity (Bolis et al., 2021). We subtracted each pairs’ lowest SRS score from their highest SRS score, a larger SRS difference score therefore indicated a greater divergence in scores and a lower score meant the pairs had similar SRS scores. Finally, we augment the quantitative results with observational case studies to examine some pairs’ interactions in further detail and to provide contextual information alongside the synchrony scores.

## Results

### SMS in Real Compared with Pseudo-dyads

**Fig. 3:**
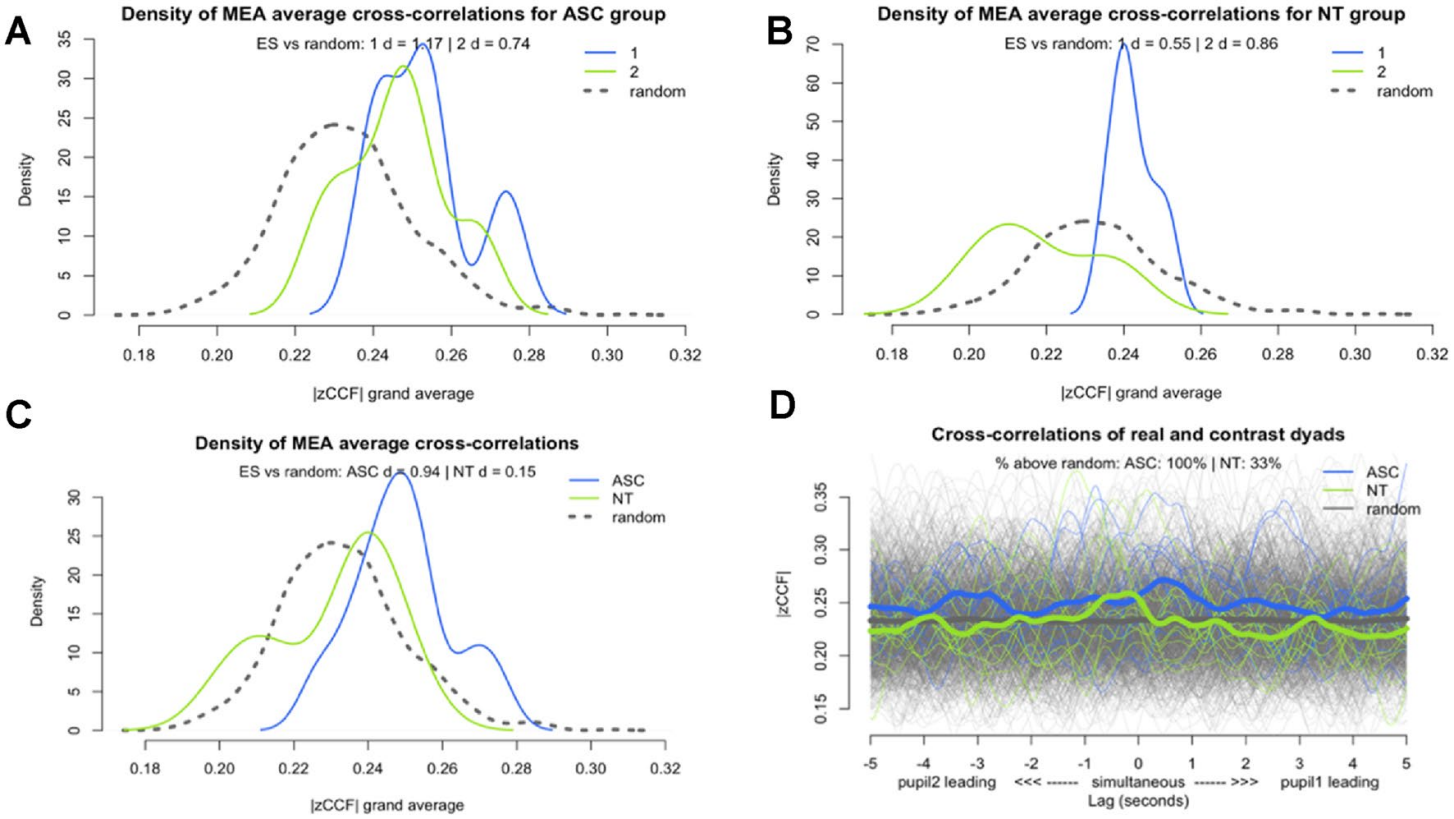
**a** Density plot of the Z transformed cross-correlation function (zCCF) for the autistic group compared to the pseudo-dyads in Colours (1) and Connect (2). **b** Density plot of the Z transformed cross-correlation function (zCCF) for the NT group compared to the pseudo-dyads in Colours (1) and Connect (2). **c** Density plot of the Z transformed cross-correlation function (zCCF) for autistic, NT, and pseudo-dyads. **d** Lag plot of Z transformed cross-correlations function (zCCF) for autistic, NT, and pseudo-dyads

We compared Social Motor Synchrony (SMS) in pseudo-dyads compared to the autistic group and NT group to examine whether the SMS displayed by each group was greater than chance. For the two activities combined, the autistic group displayed significantly higher SMS than the pseudo-dyads (*t*(10.35) = 3.87, *p* = .003, *d* = 0.94, 95% CI [0.03, 0.01]), whereas the NT group and the pseudo-dyads did not significantly differ in SMS (*t*(11.28) = − 0.54, *p* = .6, *d* = − 0.15, 95% CI [0.01, − 0.01]; see Fig. 3a). 100% of the autistic group’s cross-correlations were greater than the pseudo-dyad’s cross-correlations, but only 33% of the NT group’s cross-correlations were higher than the pseudo-dyads’ cross-correlations (see Fig. 3b).

We then compared SMS in the autistic and NT groups with pseudo-dyads in the two different activities separately. Figure 3c shows the autistic group displayed significantly higher SMS compared with the pseudo-dyads for Colours (*t*(4.06) = 3.31, *p* = .03, *d* = 1.17, 95% CI [0.04, 0.01]) but not during Connect (*t*(5.09) = 2.21, *p* = .08, *d* = 0.74, 95% CI [0.03, − 0.002]). However, the effect sizes in both activities were moderate to large. Figure 3d. shows the NT group had significantly higher SMS compared with the pseudo-dyads for Colours with a moderate effect size (*t*(5.5) = 3.76, *p* = .01, *d* = 0.55, 95% CI [0.02, 0.003]). For Connect, the NT group’s SMS was lower than for pseudo-dyads. This was not significant (*t*(5.07) = -2.33, *p* = .07, *d* = − 0.85, 95% CI [0.001, − 0.03]), but there was a large negative effect size.

### Amount of Movement in Autistic and NT Dyads

To ensure differences in SMS were not due to systematic differences between groups in movement quantity, we compared average Motion Energy in autistic compared with NT pairs. We used a rate-per-minute score to account for differing lengths of video. A Wilcoxon Rank Sum test showed the autistic (Mdn = 375.83, IQR = 288.90) and NT (Mdn = 240.08, IQR = 60.27) groups did not significantly differ (*U* = 92, *p* = .12, 95% CI [303.14, − 38.76]) in their amount of motion energy. There was also no significant association between pairs’ average motion energy and their overall SMS score (r(20) = 0.34, *p* = .11, 95% CI [0.87, − 0.56]).

### Differences in SMS in Autistic Compared with NT Pairs

**Fig. 4:**
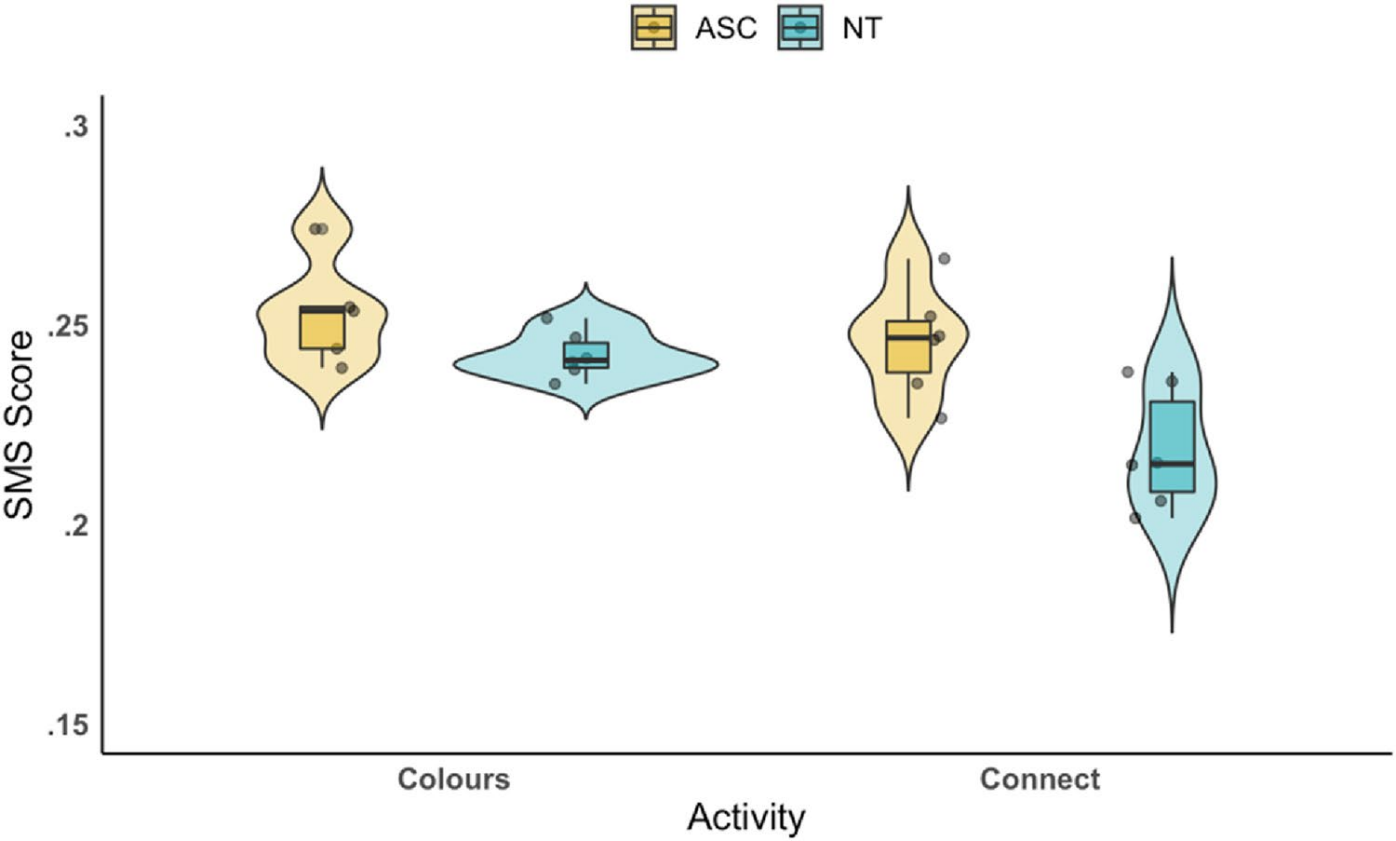
The SMS scores of autistic and NT groups in the Colours and Connect activities

We conducted a two-way ANOVA to examine differences in SMS in autistic compared with NT pairs across the two activities, and to examine within-group differences between Colours and Connect (see Fig. 4). There was a significant main effect of group (F(1,21) = 12.43, *p* = .002, *partial η*^2^ = 0.41, CI 95% [0.01, − 0.03]). The autistic group showed significantly higher levels of SMS (*M* = 0.25, *SD* = 0.01) than the NT group did (*M* = 0.23, *SD* = 0.02). There was also a significant main effect of activity (F(1,21) = 9.27, *p* = .006, *partial η*^2^ = 0.33, CI 95% [0.01, − 0.02]), with pairs displaying significantly more SMS playing Colours (*M* = 0.25, *SD* = 0.01) compared with Connect (*M* = 0.23, *SD* = 0.02). There was no significant interaction between group and activity (F(1,21) = 2.45, *p* = .13, 95% CI [0.01, − 0.04]). Post hoc tests indicated that the NT group showed more SMS during Colours (*M* = 0.24, *SD* = 0.01) than during Connect (*M* = 0.22, *SD* = 0.02), whereas the autistic group did not differ according to activity. The autistic group (*M* = 0.25, *SD* = 0.01) showed more SMS than the NT group did (*M* = 0.22, *SD* = 0.01) during Connect, but there were no significant differences between groups during Colours. However, these differences appear marginal, driven mainly by a slight reduction by the NT in the Connect activity. Also, given the confidence intervals both cross zero, we emphasise caution when interpreting the main effects.

### Associations Between SMS and Measures of Autistic Traits

**Fig. 5:**
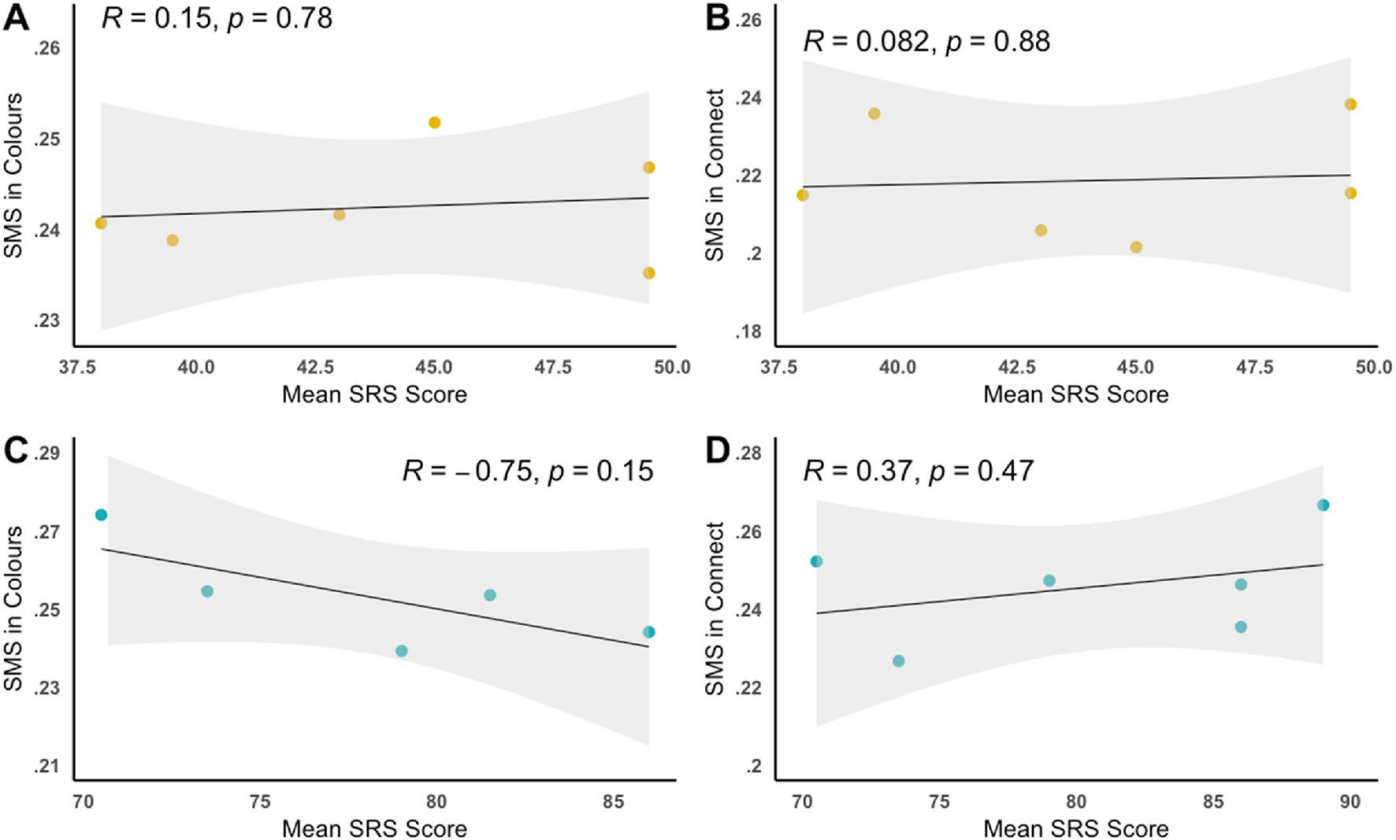
Pearson correlations with standard error between, SMS scores and mean SRS scores for the NT group in **A** Colours, and **B** Connect. SMS scores and mean SRS scores for the autistic group in **C** Colours, and **D** Connect

We collected Social Responsiveness Scale (SRS) scores for all participants and calculated the mean SRS score for each pair to examine the relationship between pairs’ combined autistic traits and SMS. The autistic group had significantly higher SRS scores than the NT group, resulting in two clusters of scores. We therefore examined the associations between SMS and mean SRS within each group separately. The NT group showed no significant associations between mean SRS scores and SMS (Fig. 5a, b) during Connect (*r*(4) = 0.08, *p* = .88, 95% CI [0.84, − 0.78]). or Colours (*r*(4) = 0.15, *p* = .78, 95% CI [0.86, − 0.75]). Similarly, the autistic group showed no significant associations between mean SRS scores and SMS (Fig. 5c, d) during Connect (*r*(4) = 0.37, *p* = .47, 95% CI [0.91, − 0.63]) or Colours (*r*(3) = − 0.75, *p* = .15, 95% CI [0.39, − 0.98]); however, there were moderate to large effects.

**Fig. 6:**
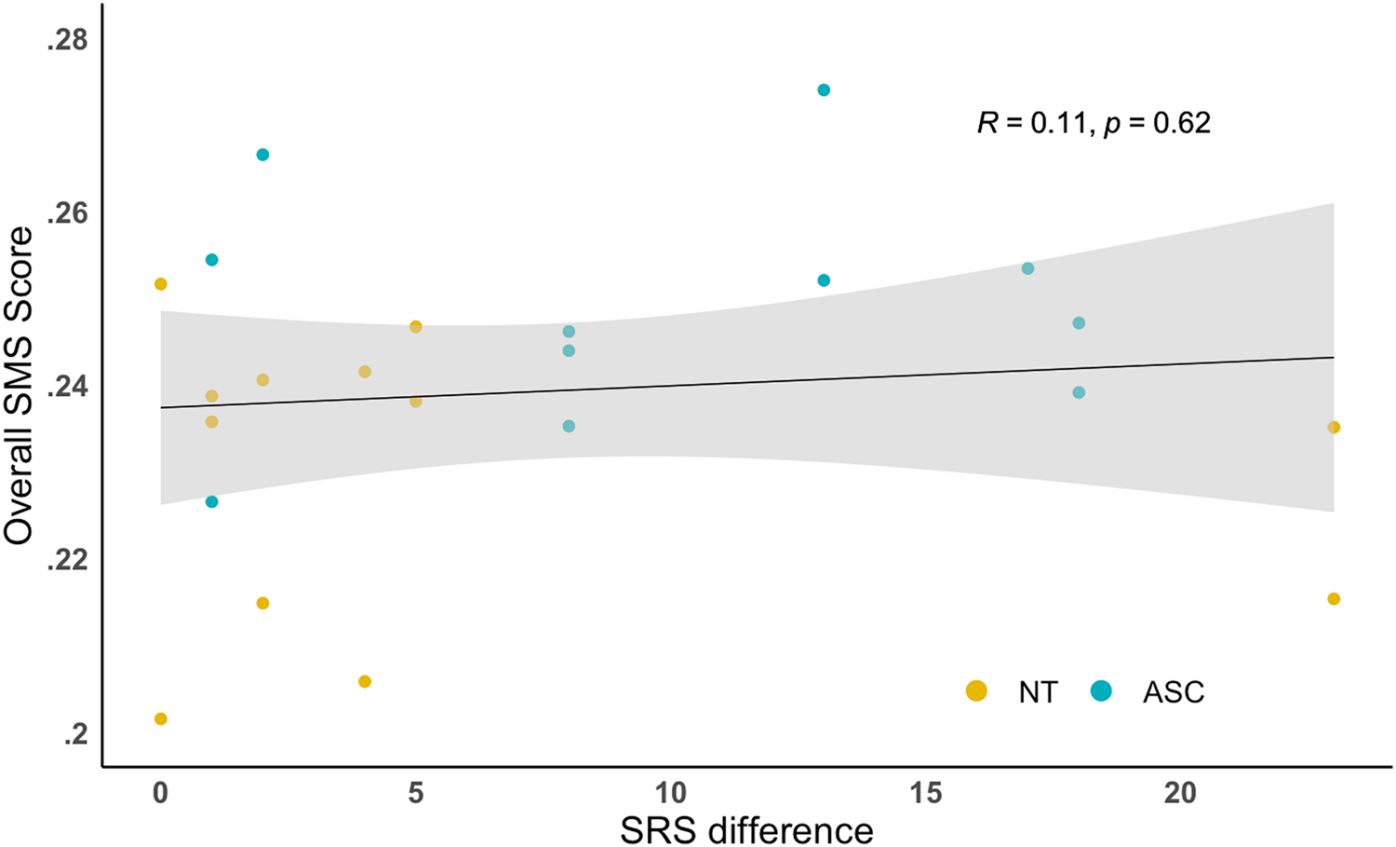
Pearson correlation with standard error between, pairs’ SMS scores and SRS difference scores

We also examined associations between SMS and an SRS difference score to investigate whether SMS scores are related to a measure of interpersonal similarity, where a higher score indicates a greater divergence in scores and a lower score meant the pairs had similar SRS scores. Figure 6 shows no association between SRS difference and overall SMS in the two groups combined (*r*(21) = 0.11, *p* = .62, 95% CI [0.50, − 0.32]). There were also no within-group associations between SRS difference and SMS in the two activities for autistic pairs (*r*(9) = 0.09, *p* = .80, 95% CI [0.65, − 0.54]), or NT pairs (*r*(10) = − 0.12, *p* = .72, 95% CI [0.49, − 0.65]). Additionally, neither group displayed significant associations between SRS difference and SMS in the Colours (ASC = *r*(3) = − 0.11, *p* = .87, 95% CI [0.86, − 0.90]), NT = *r*(4) = − 0.61, *p* = .19, 95% CI [0.39, − 0.95]) or Connect activities (ASC = *r*(4) = − 0.12, *p* = .81, 95% CI [0.85, − 0.76], NT = *r*(4) = − 0.03, *p* = .96, 95% CI [0.80, − 0.82]).

### Case Study 1: Spontaneous SMS and Rapport

**Fig. 7:**
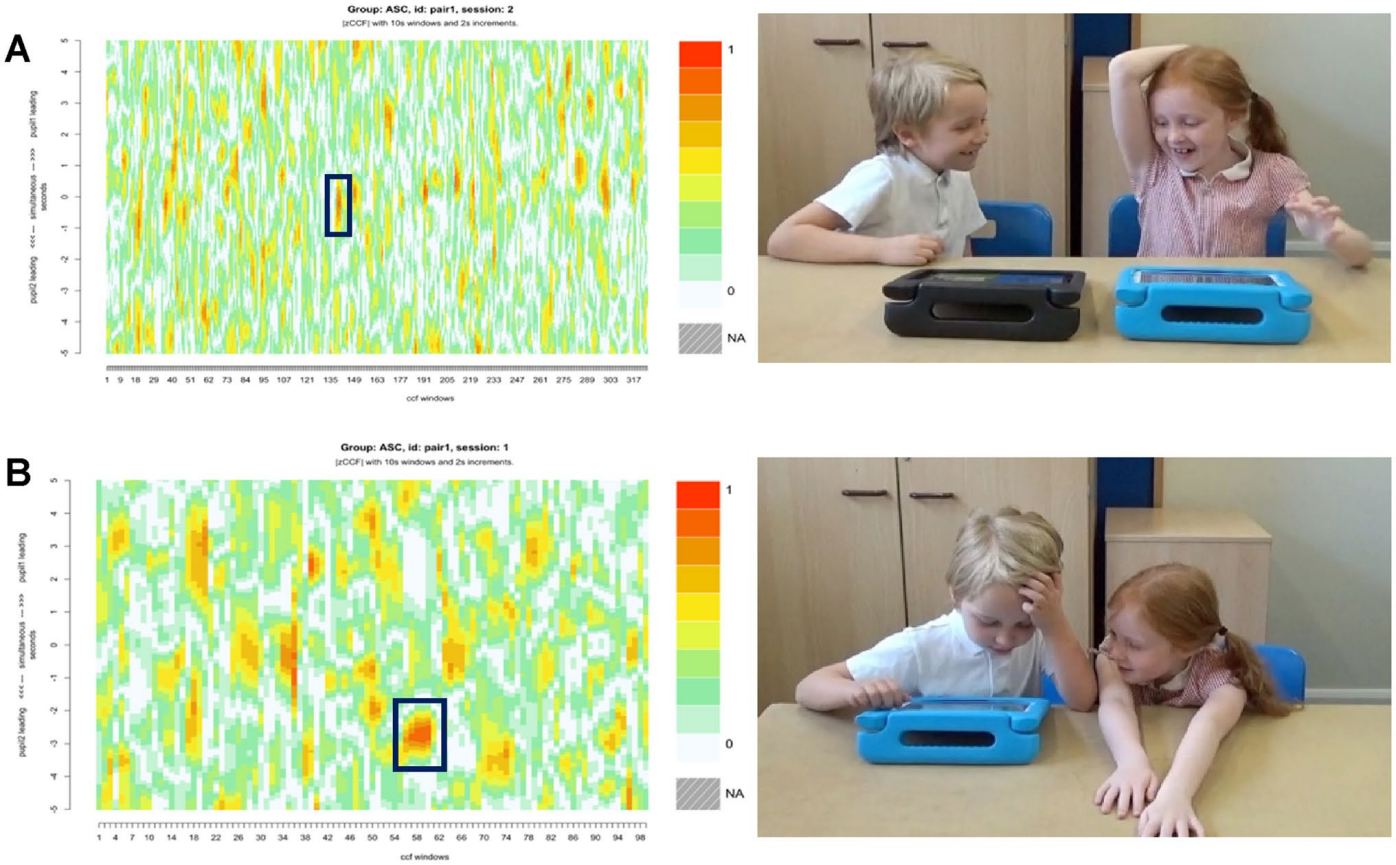
Stills and heatmaps of pair 1 (ASC) showing **a** shared enjoyment and several moments of close SMS during Connect, and **b** close SMS while partner 1 watches partner 2 play Colours. *Parental consent was granted for the use of images*

Pair 1 (autistic boy and girl) displayed the highest SMS score of either group during Colours and high levels of SMS during Connect (the second highest of all pairs). They got along well and there were moments of high energy and shared humour, particularly during Connect. The heatmap in Fig. 7a illustrates several short moments of close SMS occurring throughout the video recording. This is indicated by the red areas distributed along the x axis, which represents the video run time segmented into windows. The moments of close synchrony for pair 1 correspond to moments of enjoyment in the video. For example, the area highlighted on ‘heatmap a’ is illustrated by the still image (see Fig. 7a, right). Close analysis of the pairs’ SMS during Colours indicates synchrony in their spontaneous movement outside of the game play (i.e., not for game-related motion, such as pressing a colour). For example, in Fig. 7b partner 1 (left) is controlling the activity and partner 2 (right) is watching while showing restlessness as she waits for her turn. This moment of close synchrony is highlighted on ‘heatmap b’. The y axis shows that the lower portion of the heatmap represents instances where partner 2 led the interaction. We therefore see in this example that partner 1 joined in with partner 2’s seemingly restless motion at a slight lag.

### Case Study 2: Multi-modal SMS

**Fig. 8:**
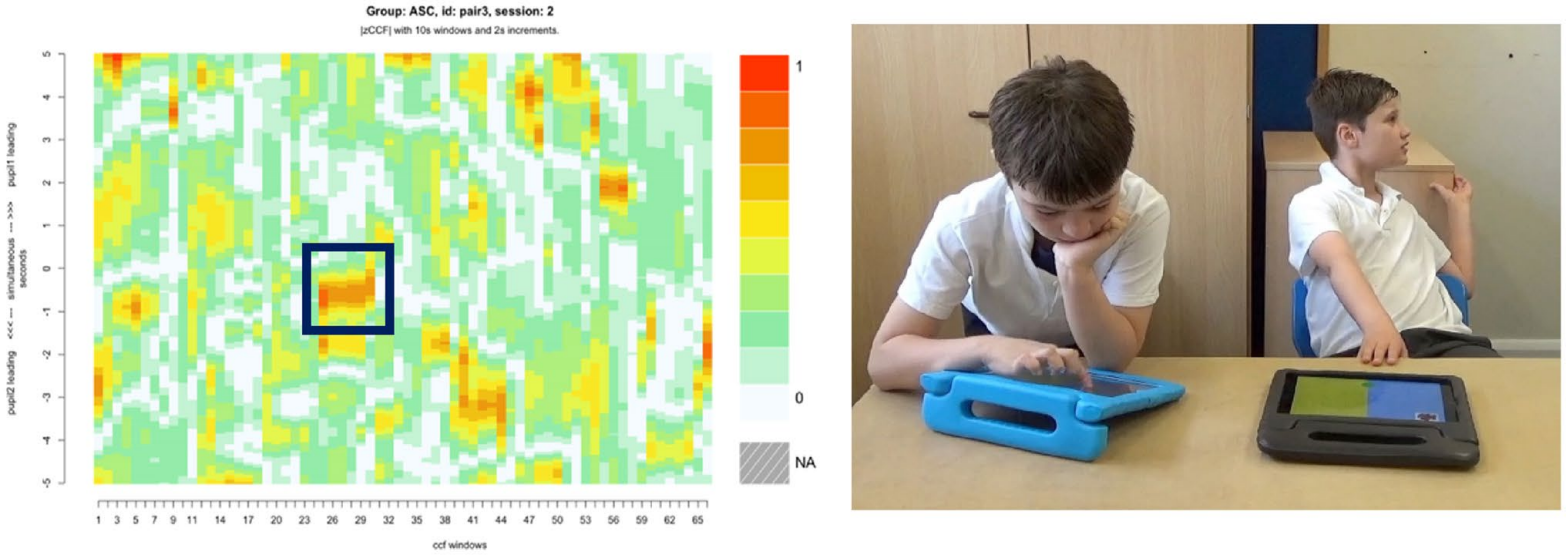
Stills and heatmaps of pair 3 (ASC) during the Connect activity, and a still of pair 3 showing close SMS in their respective hand and arm movements. *Parental consent was granted for the use of images*

For Connect, the highest SMS score was displayed by pair 3, a pair of autistic boys who showed little apparent interest in each other or the activity. However, they showed moments of close SMS, which are illustrated in the corresponding heatmap by the areas of dark red (see Fig. 8, left). During one moment of close synchrony partner 2 was disengaged from his peer and the activity. From the video, we see partner 1’s hand motion during game play was coupled with partner 2’s arm movement as he moved it across the table. This occurred with very little lag and results in a prolonged period of closely synchronised motion. This is indicated by the highlighted area appearing in the centre of the figure, which is aligned with lag 0 on the y axis. This means the pair moved synchronously and with a simultaneous onset of their movements, despite not appearing engaged with one another (see Fig. 8, right).

### Case Study 3: Negotiating a Shared Space

**Fig. 9:**
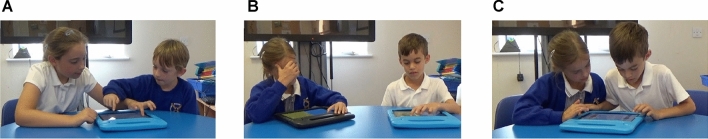
Still images of the following NT pairs: **a** pair 13 sharing their space during the Colours activity, **b** pair 8 sitting at a distance during the Connect activity, and **c** pair 8 coming together to closely negotiate a shared space during the Colours activity. *Parental consent was granted for the use of images*

Pair 13 (NT boy and girl) showed the lowest SMS in Connect but the highest of the NT group in Colours. During Colours, the pair were required to dynamically negotiate a shared space, with their movements occasionally crossing over (see Fig. 9a). During Connect, each partner remained in their own space in front of their respective iPads. Similarly, pair 8 (NT boy and girl) moved their respective iPads away from one another during Connect (see Fig. 9b). They did not verbally communicate and struggled to determine the rules of the game, resulting in the second lowest SMS displayed by all pairs. However, during Colours, both partners leaned in and attended to their partner, with higher levels of SMS than when they played Connect (see Fig. 9c).

## Discussion

We examined Social Motor Synchrony (SMS) in pairs of autistic and pairs of neuro-typical (NT) children when partners were familiar with one another, of the same neuro-type, and were matched by teachers according to their perceived compatibility. The study extended previous work examining SMS in autistic compared with NT partners, which mostly compares mixed dyads to matched-NT dyads and uses tasks that may limit SMS for autistic participants and their partners (Glass & Yuill, 2023). Notably, our findings indicate pairs of autistic children can display as much SMS, if not more, as pairs of NT children, under certain conditions. Overall, the autistic group showed slightly more synchrony than the NT group, primarily driven by higher SMS than the NT group in the Connect activity. When playing Connect, the autistic group showed higher SMS than chance, and the NT group showed SMS lower than chance, consistent with group differences. The groups showed similar levels of SMS during the Colours activity, at levels higher than chance. Considering the study’s small sample size and the confidence interval results, the differences observed between the groups should be interpreted with caution. Nevertheless, it is evident that autistic pairs may synchronise at least to an equal degree as NT pairs.

### Autistic and NT Pairs Show a Similar Degree of Movement

There were no differences between the two groups in pairs’ average amount of Motion Energy and no association between Motion Energy and SMS. This suggests the higher SMS exhibited by the autistic group during Connect is unlikely to have been a result of differences in their movement quantity. Some literature suggests autistic people move more than NT people do (Delaherche et al., 2013) However, the literature is inconsistent, with others finding no differences in movement quantity between autistic and NT groups (de Marchena & Eigsti, 2010). If the autistic group had systematically moved more, we might have understood the higher SMS in Connect compared with the NT group to be due to higher levels of average Motion Energy.

### Autistic Pairs Display More SMS in the Connect Activity than NT Pairs

For the NT group, SMS was task dependent and group differences only emerged during Connect. While these differences were slight, the NT group showed less SMS than the autistic group and lower SMS than they did during the Colours activity. The apps differ in their design and here we offer a speculation about how two core features may have produced different SMS: the dual-tablet configuration of Connect compared with the shared tablet used for Colours, and the availability for tailored content in Connect. Connect was designed from a Task-Sharing Framework (TSF), which outlines how user interfaces can mediate collaborative interactions between two or more users (Pearce et al., 2005). The dual-tablet design constrains for early collaborative behaviours, namely other-awareness and contingent action. It was designed for autistic children with learning disabilities who show little engagement with a social partner (Holt & Yuill, 2017; Yuill, 2021). We expected this design to universally support behaviours foundational for collaboration as it prompts users to attend to their partner’s workspace, encouraging pairs to approach the activity together.

We observed behaviours in the NT group that reveal how cultural norms surrounding iPad use may have affected the NT children’s approach to Connect, reducing their opportunities to synchronise. NT pairs tended to adopt solo use of their respective device. As a cultural tool, tablets are used as personal devices (Yuill et al., 2013). McLay et al. (2015) describe how iPads are used in mainstream education as tools for isolated work, and in the age of screen-time allowances, it is the norm for iPads to be used individually (Al-Jarf, 2021; Hodes & Thomas, 2021). This was reflected in our observations; NT pairs tended to pull their tablet away from their partner, which reduced their opportunity to act contingently on their partner’s gameplay. Of course, autistic children do also engage in solo iPad use. However, iPads are used frequently with autistic children in educational settings (Eden et al., 2019). They are often used as shared devices, to practice social skills such as turn-taking for example, or as communication tools (Kim & Clarke, 2015; Xin & Leonard, 2014). We observed few instances in the autistic group where they drew their iPad away from their partner, which would prevent their opportunities to collaborate. The NT group on the other hand frequently withdrew their respective devices, which appeared to prevent them working together. Since synchrony is a core component of collaboration (Roschelle & Teasley, 1995), drawing upon cultural representations of the tablet as a solo tool may have reduced collaboration in the NT children, resulting in lower SMS.

The different behaviour of NT and autistic pairs might also reflect underlying differences in mainstream and special education. It is recognised that there is an increasing emphasis on outcomes in mainstream education, which can influence the process of learning (Harris & Clayton, 2019), and may lead to a fear of failure (Szczygieł & Pieronkiewicz, 2021). Since collaboration is a vehicle for social and cognitive development (Moll & Tomasello, 2007), the process of attending and responding to one’s partner in Connect is more important than correctly categorising the pictures (Holt & Yuill, 2017). Special education often involves skills-based learning, emphasising the process as the means to the outcome (Correia & Halabi, 2021; Landrum & Kauffman, 2003). During Connect, users need to work together to determine the rules of the game. The autistic group appeared comfortable exploring the activity, whereas NT group were apprehensive in their approach. Their trepidation appeared to be a result of anxiety in showing their partner their own work in case they were incorrect, which may have contributed to the withdrawal into their own physical space. The drive towards outcomes in mainstream education may therefore have inhibited the NT children’s confidence to explore the game with their partner, reducing their opportunities to synchronise.

We personalised the Connect activity for the autistic participants as autistic people can show focused attention and engagement when tasks are meaningfully tailored to their interests (Murray, 2018; Williams et al., 2021). While some educational paradigms adopt universal design to improve accessibility (Scott et al., 2003), it is frequently recommended that reasonable adjustments are individualised (Holmes, 2014). Since it was not necessary for the content to be tailored to the NT childrens’ interests for them to collaborate, the content (sea and land animals) was designed to align with their curriculum, and to be broadly engaging for a range of children. Overall, the activity appeared challenging for the NT children. This may have been a result of an initial attempt to work in isolation as discussed, but there is also a chance the content was too challenging. If the NT children lacked confidence to make mistakes (Szczygieł & Pieronkiewicz, 2021), this could have exacerbated their tendency to withdraw from, and not synchronise with, their partner. Several of the NT children did appear to enjoy the pictures of animals, with some remarking on their different features and offering excitable vocalisations. There is therefore the possibility that SMS is related to enjoyment of the interaction with the partner and task. The lack of personalisation of Connect content for the NT children may account for the slight reduction in SMS compared with the autistic group.

### Autistic and NT Pairs Show Similar SMS in the Colours Activity

We have described the NT group’s tendency to work in isolation during the Connect activity, which may have affected their SMS. Colours on the other hand, requires partners to dynamically negotiate a shared space. Most pairs moved flexibly in and out of the shared space, each contributing to the activity and engaging in a process of fluid collaboration or turn-taking. This observation fits with the notion that synchrony is not continual or constant: people tend to fall in and out of synchrony (Mayo & Gordon, 2020), which is a dynamic and time-unfolding process (Fitzpatrick et al., 2016). The open nature of Colours requires flexible and spontaneous interaction of the kind that may support interpersonal synchrony to occur. Consequently, the open nature of the Colours activity may have fostered the type of dynamic movement to enable SMS in both autistic and NT pairs.

### The Autistic Group Showed Similar Levels of SMS in Tailored and Non-tailored Activities

We expected the autistic group to show higher SMS in the Connect activity than the Colours activity, due to the personalised content (Murray, 2018; Williams et al., 2021) and supportive dual-tablet design, which encourages complementary movements and allows simultaneous motion (Holt & Yuill, 2017). However, the autistic group showed similar SMS across the two activities. While Connect was designed to facilitate collaboration (Holt & Yuill, 2017), this scaffolded design may not be necessary for SMS. Synchrony is an important element in collaboration (Roschelle & Teasley, 1995); however, collaborative interactions are not the only social space where synchrony occurs. In fact, the current findings illustrate SMS often occurs in body movements that are not associated with direct game-play, such as in multi-modal, and micro-level movements. Additionally, most autistic pairs were engaged in both tablet activities. Even during periods when one partner dominated the shared device, the second partner tended to remain engaged by watching their partner and waiting for their turn. This supports the idea that tablet devices are highly engaging for some autistic children (Heasman & Gillespie, 2019; Mazurek et al., 2015), which may mean that personalisation was not crucial for SMS.

Nonetheless, the current findings provide some endorsement for the design of the Connect activity to scaffold SMS in some autistic pairs. There were indications of a positive relationship between SMS and Social Responsiveness Scores (SRS) in Connect, indicating high SMS in dyads with high combined autistic traits. There was also a negative relationship between SMS and SRS scores for the autistic group in the Colours activity, suggesting low SMS for dyads with high combined autistic traits. This is consistent with research examining other shared tablet activities, which suggests some autistic people can find proximity uncomfortable (Boyd et al., 2015). Sharing a device, as in Colours, may therefore be challenging for pairs with high combined autistic traits. While our small sample size may have prevented the associations reaching significance, the effect sizes were moderate to large. Taken together, this pattern of findings suggests the design of Connect is most useful in supporting SMS in peers with the highest level of social difficulty. This is consistent with the initial intention for the Connect app, which was tailored to support collaboration in autistic children with high communication difficulties (Holt & Yuill, 2017).

### The Importance of the Task and Social Context

Our findings contrast with other work showing low SMS in autistic pairs (Georgescu et al., 2020; Stoit et al., 2011). There were elements of these studies which may have affected autistic participants’ social engagement, such as additional cognitive demands, unfamiliar environments, and prescribed conversation topics. Georgescu et al. (2020) used a naturalistic paradigm and examined SMS during conversations between unfamiliar partners, with topics provided by the researchers, including ‘desert island’, meal planning, debate tasks, jokes, and role play. For neurotypical interactions, these topics may allow enough flexibility to be engaging and foster a social connection between strangers. However, we know autistic people can communicate differently from NT people and can communicate better when both topics and conversational partners are preferred (Sturrock et al., 2021). Different conversational devices are used to achieve intersubjectivity in pairs of autistic people, such as abrupt topic changes, which could be limited given prescribed conversation topics (Heasman & Gillespie, 2019).

Social interaction can be challenging for some autistic people, particularly when environments and tasks are stressful or overwhelming, or when interacting with non-autistic people (Crompton et al., 2020c). They can take more time to habituate to new social environments (Vivanti et al., 2018), can have executive functioning differences, particularly in unfamiliar and sensorily demanding environments (Craig et al., 2016), and can find it easier to engage when tasks are meaningful (Williams et al., 2021). We used tasks tailored to the needs and interests of autistic participants, and partners who were familiar with one another and with the study environment. We also matched the task difficulty to the abilities of each pairs using teachers’ advice in anticipation of task difficulty as a potential factor affecting synchrony. These adaptations may have allowed the autistic pairs to synchronise as well as NT pairs. Our results provide an indication of the potential for SMS in autistic pairs to match SMS in NT pairs when the tasks and social environment are considered in relation to autistic participants’ needs. The findings therefore suggest modifications to an SMS model of autism (Fitzpatrick et al., 2016): when potential barriers for embodied social interaction are removed, such as executive function demands, autistic pairs may synchronise as well as NT pairs.

### Interpersonal Similarity

We drew upon theories of Dialectical Misattunement and Double Empathy (Bolis et al., 2018; Milton, 2012), and literature suggesting matched-neurotype interactions feel more comfortable and connected than mixed-neurotype interactions (Crompton et al., 2020b, 2020c). We therefore expected SMS to be similar in pairs of autistic and pairs of NT children. The groups showed similar levels of SMS in the Colours activity, and the autistic group displayed more SMS than the NT group during Connect. We have discussed the potential difficulty of the NT group with the Connect activity. However, the groups’ similarity of SMS during Colours suggests autistic children can synchronise to a similar degree to their NT peers when tasks and contexts are suited to their needs. To ascertain whether interpersonal similarity facilitates SMS, it is necessary to compare dyads of the same neuro-type with mixed dyads under these conditions. However, these findings provide initial evidence of the potential for autistic peers to synchronise as well as NT peers.

To investigate the impact of interpersonal similarity on SMS, we computed an SRS difference score, where a higher score represents dissimilarity of autistic traits. Bolis et al. (2021) found an association between similarity of autistic traits and perceived closeness with one’s partner. In this study, there were no significant associations between SRS difference and SMS for either group in either activity. This might suggest SMS is achievable in autistic interactions when partners have diverse interactional styles when the context is carefully considered. However, a similarity of one’s disposition likely goes beyond autistic traits. We saw the highest SMS in pair one (ASC), for instance, whose SRS difference score was in the highest 50% of the sample, suggesting they have markedly different social abilities. However, this pair showed excellent rapport and had a strong bond. As SMS is associated with affiliation and social connection (Hove & Risen, 2009; Tuncgenc & Cohen, 2016), it is unsurprising that we see the highest SMS in the most connected relationships. We note autistic people can take longer than NT people to feel comfortable in new social interactions (Vivanti et al., 2018), yet previous literature involves autistic pairs in transient interactions between unfamiliar partners (Georgescu et al., 2020; Stoit et al., 2011). Partnering participants according to their perceived disposition and relationship in the current study may therefore have supported SMS.

Close SMS has been shown to represent stronger social bonds and even better therapeutic outcomes (Nyman-Salonen et al., 2021; Tuncgenc & Cohen, 2016). However, we know that more SMS is not always better. Mayo and Gordon (2020), for instance, argue that moving in and out of synchrony demonstrates an adaptive and flexible interpersonal system. In this study, pair three showed the highest SMS in Connect despite appearing socially disconnected. However, when comparing their heatmap (see Fig. 7) with the heatmap of pair one (see Fig. 6) who had the highest SMS scores overall, we see different SMS patterns. Pair three’s interaction was characterised by stronger moments of synchrony, shown by the larger red areas on the heatmap, but these were few and sporadic. The large amount of white on the heatmap also suggests weak SMS connectivity overall (see Fig. 7). Pair one on the other hand, had consistent bursts of SMS throughout the activity, suggesting a more flexible and adaptive interaction (see Fig. 6). Wan et al. (2022) suggest other broader elements of IPS, namely coordination and contingency, relate to perspective taking but synchrony does not. It is therefore likely that SMS alone does not represent smooth and connected interactions, and broader elements of IPS interact to facilitate social attunement.

### Limitations

Recent literature has studied several aspects of interpersonal synchrony (IPS) in isolation, including Social Motor Synchrony (SMS). However, SMS is one layered element of IPS, which goes beyond rhythmically-matched body movements. IPS includes several additional layers of communication and is therefore difficult to concretely define and measure in its entirety (Lambrechts et al., 2014; Zamm, 2018). Other elements of IPS may be different in interactions involving an autistic person compared with two NT people, such as imitation (Vivanti & Hamilton, 2014). Additionally, synchrony can be co-expressive and occur between different modalities (Lambrechts et al., 2014; Loehr, 2012). Ward et al. (2018) demonstrated how autistic children can show synchrony that does not immediately appear socially relevant, such as a child’s stimming motion coupled to the body movements of drama facilitators. This highlights the importance of concurrently examining several types of IPS. Autistic partners may synchronise across modalities in unexpected channels when looking at interaction through a neurotypical lens.

Our study enabled the investigation of SMS in a sample of autistic children who are frequently excluded from research owing to their difficulty with verbal communication. Tailoring the tasks and contexts to the needs of these participants shows the potential for inclusive synchrony research. While the current research restricts the measurement of IPS to SMS, it is a step towards better understanding the synchrony that is possible between autistic people and their interaction partners, which may depend on the task and context. Still, our findings are provisional due to the small sample size. The sample size limitations are compounded as three autistic pairs did not complete both activities, thus reducing the sample size further. While it is comparable to other research involving pairs of autistic children in SMS research (e.g., Fulceri et al., 2018), further replication is needed due to power limitations. Finally, during this study, teachers paired children according to their perceived disposition. This evidently influenced the gender composition of pairs in the NT group, as all were mixed gender. This contrasts with the autistic group, which included only one mixed gender pair. The remaining pairs in the autistic group comprised two boys. This reflects the gender skew in identification of young people with special educational needs in the UK; in 2021, 73.1% of pupils with an Education Health Care Plan were boys (Department for Education, 2022). Some literature indicates IPS is higher in same-gender partnerships, with female dyads displaying higher SMS than male dyads (Cheng et al., 2017; Feldman, 2003). However, females have been shown to have high levels of interpersonal sensitivity, which may enable greater synchrony than in pairs of two males, as with most of the autistic group in the current study (Hall et al., 2006). We also note that the pair with the highest SMS in this study was a mixed gender pair. Still, a more controlled matching of participants in synchrony research on measures that may influence IPS will allow more accurate investigation into any differences present in IPS between autistic and NT pairs and allow further examination of a SMS model of autism.

### Conclusion

Our findings add to the growing literature surrounding Interpersonal Synchrony (IPS) in autism. It is one of few studies so far that examines Social Motor Synchrony (SMS) in pairs of autistic participants. This is an important addition to the literature considering reports of close and connected relationships between autistic people, and the evident association between affiliation and IPS. In carefully tailoring the activity to the needs and interests of autistic participants, we demonstrate how SMS in autistic pairs can equal the SMS of neuro-typical (NT) pairs. NT pairs showed lower SMS than autistic pairs in the Connect activity, which was more suited to the needs of the autistic pairs and their approach to the tablet device. Notably, the NT pairs’ difficulty with Connect demonstrates the influence of the type of task on the SMS for both NT and autistic participants, as we have identified in some previous literature. We therefore advocate a careful selection of SMS tasks in future and stress the need for more SMS research in natural environments, such as classrooms or during free-play interactions in familiar and comfortable environments. While these findings are limited given the sample size and potential influence of gender, we demonstrate the potential for autistic partners to synchronise, which requires modification to the idea of a synchrony deficit in autism. We also demonstrate the possibility for including autistic participants who are pre- or minimally-verbal in synchrony research, highlighting their behaviour as socially relevant and connected.

## Footnotes

1 Education and Health Care Plans (EHCPs) are legal documents, which detail a child’s special education needs based on assessments by multidisciplinary teams including health and education professionals.
